# Promoting Probiotics Survival by Microencapsualtion with Hylon Starch and Genipin Cross-linked Coatings in Simulated Gastro-intestinal Condition and Heat Treatment

**Published:** 2018

**Authors:** Mohammad Ali Khosravi Zanjani, Mohammad Reza Ehsani, Babak Ghiassi Tarzi, Anousheh Sharifan

**Affiliations:** *Department of Food Science and Technology, Science and Research Branch, Islamic Azad University, Tehran, Iran.*

**Keywords:** Genipin cross-linking, Chitosan, Poly-L-lysine, Hylon, Simulated gastro-intestinal condition, Heat treatment

## Abstract

Microencapsulation with hydrocolloids as a modern technique has been used to prolong the survival of probiotics during exposure to harsh conditions. In this study, alginate-Hylon starch microcapsules with genipin cross-linked chitosan and poly-L-lysine coatings were developed to encapsulate four strains of probiotic bacteria, including *Lactobacillus casei* (ATCC 39392), *Bifidobacterium bifidum* (ATCC 29521), *Lactobacillus rhamnosus* (ATCC 7469), and *Bifidobacterium adolescentis* (ATCC 15703). The viability of probiotics was investigated under heat treatment (72, 85, and 90 °C, 0.5 min), simulated gastric juice (along with pepsin, pH = 2, 2 h at 37 °C), and simulated intestinal juice (along with pancreatin and bile salts, pH = 8, 2 h at 37 °C). The morphology and size of microcapsules were measured by scanning electron and optical microscopy. Results of this research demonstrated that, compared with the free form, microencapsulated probiotics had significantly (*P* < 0.05) higher viability under simulated gastro-intestinal conditions and heat treatment. Microcapsules with genipin cross-linking significantly increased the viability of probiotics compared with non-cross-linked microcapsules. Moreover, genipin did not influence the size of the microcapsules produced using the emulsion technique. In general, this research indicated that the presence of genipin as a cross-linking agent in the structure of hydrocolloids such as chitosan and poly-L-lysine, and also the presence of Hylon (high-amylose starch) as a material resistant to heat and digestive enzymes, not only increased the viability of probiotics in simulated human gastro-intestinal condition but also considerably improved the thermal resistance of microcapsules.

## Introduction

Probiotics are defined as live micro-organisms that confer a health benefit to consumers when administered in adequate amounts (1). Bacteria belonging to the genera *Bifidobacterium *and *Lactobacillus *are often used as probiotic supplements (2). The necessary conditions for the beneficial effects of probiotics to be realized are that they should be active and alive upon reaching the intestine (3–5). In addition to their mutation suppressive and anticancer properties, probiotics bestow other health benefits on their hosts, such as maintaining the natural intestinal microflora, improving the immune system, reducing lactose intolerance, and lowering blood cholesterol levels (1, 6–9). According to current standards, there must be 10^6^ to 10^7^ CFU of these probiotics per gram of probiotic foods and drugs for them to have beneficial health effects (1). To promote the viability of probiotic bacteria under harsh conditions, various techniques have been used to prolong their survival (3, 10–15), one of the newest of which is microencapsulation. This technique consists of protecting microorganisms with a layer of hydrocolloids on a microscopic scale to encapsulate and separate them from their environment, thereby increasing their survival under adverse conditions (12, 13, 16–18).

Alginate is a linear heteropolysaccharide that is extracted from various algae. Its building blocks are D-mannuronic acid and L-glucuronic acid joined together by glycosidic linkages. The simple technology, ease of use, non-toxicity, low price, and the fact that it is known as a safe food additive, are among the advantages of using alginate for microencapsulation. Alginate capsules can be produced using emulsion and extrusion methods (15, 19–22). Moreover, mixing calcium alginate with starches from different sources creates a coherent and uniform structure in addition to prolonging cell survival due to some of the prebiotic properties. A variety of starches and modified starches as a filler material have been tested to encapsulate probiotic bacteria (22–26). Starches create an integrated and uniform mixture with alginate matrix because of their well binding capacity and adequate solubility and this, in turn, facilitates capsule formation and efficiency in the microencapsulation procedure (13, 22-28). Hylon maize starch granules have a high ratio of surface area to mass. Their high amylose content leads to greater resistance of Hylon starch granules against high temperature and digestive enzymes compared with low amylose starches, such as wheat or corn (10, 13, 20, 27-31).

Because the formation of calcium alginate gel occurs in the presence of calcium ions, the presence of singly charged ions (due to ionic competition), and chelating agents of calcium ions, such as phosphates, acetates, and citrates, leads to the destruction of calcium alginate capsules Furthermore, alginate is generally very sensitive to low pH values and heat and loses its structure very easily under these conditions (10–14). However, encapsulation of alginate capsules, which are negatively charged, with chitosan and poly-L-lysine creates coated microcapsules, enhances their physical and chemical stability, and reduces the destructive effects of chelating agents and substances that chelate calcium ions present in the structure of the microcapsules (13, 32–37). The polycationic nature of chitosan leads to a strong interaction of carboxylic groups of alginate with the amine groups of chitosan, resulting in the formation of a membrane (13). Many results have been reported concerning the use of chitosan-coated microcapsules as a drug delivery formulation (34–38). Poly-L-lysine is similar to chitosan in that it forms a strong complex with alginate and thereby strengthens alginate microcapsule membranes. The presence of free amines in poly-L-lysine molecules allows the formation of various bioproducts, such as proteins, sugars, and peptides that bind to poly-L-lysine by simple chemical interactions (32). This allows the use of poly-L-lysine as a carrier of drugs and in gene therapy using nanoparticles as well as the employment of liposomal forms of poly-L-lysine (39). Furthermore, the formation of multilayered covers of ploy-L-lysine and alginate was attempted to form multicoated microcapsules (32, 39–43). To further increase microcapsular thermostability and resistance to enzyme degradation, chitosan and poly-L-lysine coatings can be chemically cross-linked by different cross-linkers (44). This reaction leads to the formation of a three dimensional (3D) network in the structure of microcapsules (45). However, most synthetic cross-linkers, such as glutaraldehyde or epoxy compounds, have the recognized disadvantage of potential cytotoxic effects and are not preferable in the case of probiotics or live-cell microencapsulation (46–50). Genipin is an iridoid glucoside, non-toxic, natural cross-linker extracted from gardenia fruits. Genipin, as one of the most important natural cross-linkers, has a remarkable effect on the stability of polymers with primary amino groups such as chitosan, collagen, gelatin, and poly-L-lysine. Genipin, with a wide range of properties, can be used for tissue fixation, membrane reinforcements, and tissue engineering (46–55). Furthermore, genipin reveals remarkable effects as an anti-inflammatory and antiangiogenic agent and has been approved for food-grade and pharmaceutical use in Japan, Taiwan, Korea, and other South-East Asian countries (48–55). The use of multilayered capsules and the presence of genipin as a cross-linking agent can play a substantial role in improving the structure of the capsules, resulting in longer survival periods of probiotics.

The main purpose of the present research is to prepare and characterize a novel coated microcapsule formulation using chitosan, poly-L-lysine, genipin cross-linking, and Hylon as a filler material for the first time. No studies have yet been reported using the technique of microencapsulation by these novel coatings in order to verify the possibility of promoting the viability of *Lactobacillus casei* ATCC 39392, *Bifidobacterium bifidum* ATCC 29521, *Lactobacillus rhamnosus* ATCC 7469, and *Bifidobacterium adolescentis* ATCC 15703. Furthermore, this study was undertaken to asses the survival of probiotics under simulated gastro-intestinal conditions (along with pepsin and pancreatin) and high temperature processes to uncover information that could be important given the health benefits of using probiotics in pharmaceutical and food materials and test its feasibility for potential biomedical applications. It must be mentioned that investigating the survival of probiotics with these novel coatings in heat treatment is also a new innovation of this study, because probiotics may be subjected to heat processes (such as pasteurization and sterilization) in food or pharmaceutical products before gastro-intestinal conditions.

## Experimental

*Preparation of cell suspension for microencapsulation*

Four probiotic strains, *Lactobacillus casei* ATCC 39392 (American Type Collection Cultuture), *Bifidobacterium bifidum* ATCC 29521, *Lactobacillus rhamnosus* ATCC 7469, and  *Bifidobacterium adolescentis* ATCC 15703 in purified and lipophilized form were purchased from the collection at the Iran Scientific and Industrial Organization.* Lactobacillus *and *Bifidobacterium *strains were inoculated in MRS broth (de Man-Rogasa-Sharpe) for 24 h under aerobic and anaerobic conditions at 37 °C, respectively. The anaerobic conditions for *Bifidobacteria* were provided by using an anaerobic jar and the gas-pack system. The biomasses were then harvested by centrifuging at 4000 rpm for 10 min at 4 °C. The cultures were then washed twice by sterile saline solution (0.9%) and used in the microencapsulation process (3, 13).

*Microencapsulation of probiotics*

Microcapsules were produced using encapsulation method reported by Khosravi-Zanjani *et al*. (13) in 2014 and Donthidi *et al*. (20) in 2010. Two percent high amylose starch (Hylon VII, Maize starch, National Starch & Chem. Ltd., Manchester, UK) was separately gelatinized first and, after it was cooled, alginate (Sigma-Aldrich 71238) was slowly added to it, and this solution was then mixed with the microbial suspension for 5 min. The obtained mixture was added to 500 milliliters of corn oil that contained 0.2% Tween 80 emulsifier and was dispersed for 20 min using a magnetic stirrer to form a uniform emulsion (350 rpm, Heydolph Stirrer, Germany). To form the microcapsules, 0.1M calcium chloride was added to the solution. After 30 min, when the microcapsules precipitated, a decanter and centrifuge at 350 g was used to separate them, and the separated microcapsules were washed with distilled water and kept at 4 °C. 

*Coating the microcapsules with chitosan*

Chitosan coating procedure was adapted from Khosravi-Zanjani *et al*. (13) in 2014 and Krasaekoopt *et al. *(32) in 2006. Chitosan (Low molecular weight, Sigma-Aldrich 448869) was dissolved in 90 mL distilled water acidified with glacial acetic acid to achieve a final chitosan concentration of 0.4% (w/v). The pH was then adjusted to between 5.7 and 6 by adding 1 m NaOH. The mixture was filtered using filter paper (Whatman NO. 41) and sterilized in an autoclave at 121 °C for 15 min. The alginate microcapsules from the previous step (15 grams) were dispersed in this solution at 200 rpm for 1 h for the coating operation to be thoroughly carried out. The microcapsules, which were coated with chitosan, were separated using a centrifuge at 350 g and, finally, they were washed with physiological serum and kept in a 0.1% peptone solution at 4 °C.

*Coating the microcapsules with poly-L-lysine*

Poly-L-lysine aqueous solution was prepared according to Constantinidis *et al*. (40) in 2007 Krasaekoopt *et al. *(56) in 2004. Fifteen grams of the sodium alginate microcapsules were added to 100 mL of a 0.05% solution of poly-L-lysine solution (Low molecular weight, Sigma-Aldrich L8995) and stirred at 100 rpm for 1 h at ambient temperature for the coating operation to be thoroughly carried out. The microcapsules, which were coated with poly-L-lysine, were separated using a centrifuge at 350 g and, finally, were washed with physiological serum and kept in a 0.1% peptone solution at 4 °C. 

*Preparation of coated microcapsules cross-linked with genipin*

For the formation of cross-linked microcapsules, and for strengthening chitosan and poly-L-lysine structure, microcapsules coated with chitosan and poly-L-lysine (15 grams) were put in a genipin solution (2.5 mg/mL, Challenge Bioproducts Ltd., Taiwan) individually and stirred slowly at 200 rpm for 18 h at room temperature for the cross-linking to be thoroughly carried out. The cross-linked microcapsules were then separated using a centrifuge at 350 g and, finally, were washed with physiological serum and kept in a 0.1% peptone solution at 4˚C (46, 47, 55). 

*Study of the survival of probiotics under simulated gastric and intestinal juices and inoculation of cells*

Simulated gastric juices according to the Brinques *et al.* (3) method in 2010 were prepared by dissolving pepsin (Sigma-Aldrich P7000) in sterile sodium chloride solution (0.5%, w/v) to a final concentration of 3.0 g/L and adjusting the pH to 2 with hydrochloric acid. According to Chavarri *et al.* (14) in 2010 method simulated intestinal juices were prepared by suspending pancreatin (Sigma-Aldrich P-1500) in sterile sodium chloride solution (0.5%, w/v) to a final concentration of 1 g/L, with 4.5% bile salts (Oxoid, Basingstoke, UK) and adjusting the pH to 8.0 with sterile NaOH (0.1 M). Both solutions were filtered for sterilization through a 0.22 μm membrane. The probiotic bacteria were inoculated to the simulated gastro-intestinal juice individually in five different forms, non-encapsulated, encapsulated with alginate coated with poly-L-lysine, alginate coated with chitosan, alginate-poly-L-lysine cross-linked with genipin, and alginate-chitosan cross-linked with genipin. It should be noted that Hylon starch exists merely in the encapsulated forms as a protective and filler compound. Then one gram of freshly encapsulated bacteria samples or 1 mL of cell suspensions (free cells) were gently mixed with 10 mL of sterile simulated gastric juice or sterile simulated intestinal juice and incubated at 37 °C for 30, 60, 90, and 120 min. Surviving bacteria were numerated by pour plate counts in MRS agar aerobically incubated at 37 °C for *lactobacillus* strains and in MRS agar anaerobically incubated at 37 °C for *bifidobacterium* strains for 2 days.

*Study of thermal stability of the microcapsules*

One gram of the produced microcapsules was added to 10 mL of distilled water in sterile test tubes to study thermal stability of the microcapsules. The test tubes were exposed to 72, 85, and 90 °C for 0.5 min and were then rapidly cooled using chilled water and the bacteria were counted using the mentioned method for three replicates (57).

*Counting bacteria entrapped in the microcapsules*

 A sodium citrate buffer and a stomacher instrument (Funk Gerber, Germany) were used to count bacteria entrapped in the microcapsules. One gram of the microcapsules was mixed with 9 ml of a sterile sodium citrate buffer (0.1M at pH: 6.3), and the mixture was transferred under sterile conditions to the bags of the stomacher instrument (the bags were autoclaved beforehand) until bacteria released from microcapsules completely. The counts (CFU/g) were determined by plating on MRS agar plates and incubating for 48 h at 37 °C. *Lactobacillus* and *Bifidobacterium* strains were cultured under aerobic and anaerobic conditions, respectively and the free bacteria were treated similarly. All samples were counted in triplicates. It is important to note that the viability of microencapsulated probiotics in sterile sodium chloride solution (0.5%, w/v) and peptone water was also measured at 4 °C for three months. The microcapsules were dissolved in the appropriate buffer solution after three months and they were used to determine the total number of viable cells (3, 13).

*Size and morphology of microcapsules*

 The mean diameter of microcapsules was measured by optical microscopy using a Motic BA300 light microscope. The diameters of 100 randomly selected capsules were measured using measurement software (Leica Qwin 550). A scanning electron microscope (LEO 440 I, England) was employed for the accurate identification of the surfaces and morphology of the microcapsules. The capsules were placed on a specimen aluminum stub with the help of double- sided sticky tape and coated using a sputter coater for 2 min at an accelerating voltage of 15 kV (15).

*Microencapsulation yield*

 The following relation was used to calculate the percentage of probiotics entrapped in the capsules (microencapsulation yield):

EY = (N/N0) × 100

In the above relation, EY is the microencapsulation yield, N the number of microencapsulated bacteria per gram of capsules, and N0 the number of bacteria in the initial microbial suspension.

*Statistical analysis*

The experiments were analyzed using a factorial experiment in full accidental state with three repetitions using *SPSS 20 Statistics* software. The obtained results were compared by Duncan›s new multiple range test.

## Results and Discussion

*Morphology and size of microcapsules and microencapsulation yield*

The size and encapsulation efficiency of microcapsules are shown in Table 1. No significant difference was observed between different microcapsules (*P* > 0.05). Moreover, the outcomes also showed that probiotics were effectively entrapped using gentle approaches. Our findings indicated that the average yield of all samples after encapsulation, coating and cross-linking was 97.67 %. These findings are in agreement with those of Mokarram *et al*. (15) in 2009 Khosravi-Zanjani *et al. *(13) in 2014. Furthermore, the viability of probiotics in sterile sodium chloride solution (0.5%, w/v) and peptone water at 4 °C showed that the number of bacteria in all the samples (microencapsulated forms) remained significantly unchanged and genipin as a cross-linker had no adverse effect on probiotics viability. This finding is in agreement with those of Annan *et al*. (52) in 2008, Chen *et al*. (49) in 2010, and the similar study done by Borza *et al*. (51) in 2010 who reported the presence of genipin in microcapsules or increasing the genipin concentration significantly increased the physical stability of microspheres without having an adverse effect on cell viability. Moreover, different studies have shown that genipin has been found not to be cytotoxic to human cells and is widely used in herbal medicine in Asia and in the fabrication of food dyes (48-55). Genipin has been found to be far less cytotoxic than many cross-linking agents used in the manufacture of biomaterials and pharmaceutical materials and this may also explain the recent increase in interest for its use (48-55).

**Figure 1:**
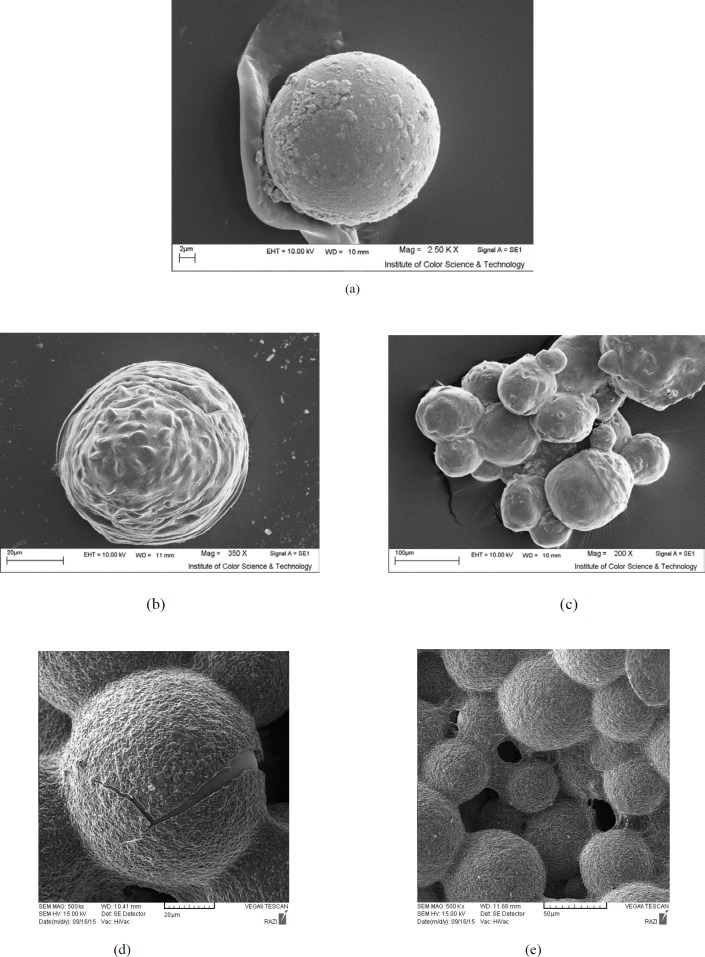
Scanning electron photomicrograph of microcapsules showing a) Alginate without coating, b) Alginate coated with chitosan, c) Alginate coated with poly-L-lysine, d) Alginate-poly-L-lysine cross-linked with genipin, e) Alginate-chitosan cross-linked with genipin

Scanning electron microscopy revealed that the formed microcapsules were all spherical and uniform. The material and type of hydrocolloid used as a coating in the formation of each microcapsule had distinct and influential effects on the appearance of the microcapsules. Moreover, the presence of the chitosan and poly-L-lysine layer, changed the surface and morphology of microcapsules and created a smooth and uniform surface for microcapsules (Figure 1). The images of microcapsules taken by the electron microscope are completely similar to those prepared by other researchers who used the emulsion technique to produce calcium alginate capsules together with other hydrocolloids (6, 10, 11, 16, 17, 20, 21). Khosravi-Zanjani *et al*. (13) in 2014 reported that the presence of chitosan changed the morphology and shape of the produced microcapsules in addition to increasing their size. However, corn starch used as filler had no effect on the size of the microcapsules.

As can be clearly seen in the electron microscope images, capsules containing genipin seemed more spherical and uniform and were structurally more coherent and stronger than capsules that were without genipin (Figure 1). Since genipin is only a cross-linker and does not increase the size of the microcapsules as a separate coating, its presence did not increase the size of chitosan and poly-L-lysine coated microcapsules but rather created structural order and cohesion in the microcapsules and improved their strength. The size of microcapsules was analyzed by measurement software (Leica Qwin 550). The mean diameter of chitosan-coated and poly-L-lysine microcapsules was 117 ± 2.96 μm and 113 ± 3.01 μm respectively. Genipin changed the morphology and shape of microcapsules obviously, and improve the spherical structure of microcapsules without increasing the size of microcapsules. In agreement with our findings, Annan *et al*. (52) in 2008, Chen *et al*. (50) in 2010, and Borza *et al*. (47) in 2007 found that the amount of genipin used as a cross-linking agent and increasing genipin concentration in the preparation of gelatin and alginate microcapsules did not significantly influence the size distribution of the beads. In this technique, the capsules are formed in micron range size. Microcapsules with double coating sodium alginate used in Mokarram *et al*. (15) study in 2009 were also micron size range (75.339 ± 0.209 μm). Various studies have indicated that a decrease in the microcapsule size to smaller than 100 μm would not suggest an important rise in the rate of probiotic survival in simulated human gastric juice (3, 24, 58). However, very large capsules can leave sandy and other undesirable textural properties to functional foods. In addition, the smaller sizes and spherical shape of the capsules cause fewer changes in the product and prevent a sand-like texture in sensory evaluation of the final product (6, 10, 11, 16).

*Survival of free and microencapsulated probiotics in simulated gastric juice*

As shown in Figures 3 and 4, viability of probiotics decreased significantly under simulated gastro-intestinal conditions *(P *< 0.05). After 120 min, viability and survival of free (non-encapsulated) *Lactobacillus* strains (*L.casei and L.rhamnosus*) decreased under simulated gastric juice conditions by 8.3 and 8.4 log cycles, respectively (Figure 3), while viability of microencapsulated *Lactobacilli* increased significantly *(P* < 0.05). Chitosan and poly-L-lysine coatings significantly improved viability of all the studied probiotics. As is shown in Figure 3, it must be mentioned that no significant differences were observed between viability of *L.casei* and *L.rhamnosus* strains in the various tested states. This also held true for the various tested *Bifidobacteria* (i.e., *B.bifidum* and *B.adolescentis*) strains. Although chitosan and poly-L-lysine coatings significantly increased survival rates of probiotics, no significant differences were observed between them with respect to viability of the probiotics. In other words, viability of probiotics microencapsulated by these two coatings was similar. However, viability of probiotics in genipin cross-linked microcapsules rose to a higher level compared to capsules without cross-linking. Presence of genipin as a cross-linking agent in the structure of the capsules significantly enhanced their viability after 120 min (by about 2.04 log cycle for the *Lactobacilli* and by approximately 2.14 log cycles for *Bifidobacteria* as compared to capsules without cross-linking). Comparison of the viability of the tested probiotics suggested that *Lactobacilli* had higher survival rates in both their free and microencapsulated forms as compared to *Bifidobacteria *because of their greater resistance to acidic conditions as against the low acid resistance of *Bifidobacteria*. The numbers of both tested strains of free *Bifidobacteria* significantly decreased under simulated gastric conditions so that after being incubated for 120 min their numbers dropped to almost zero. This finding is in agreement with those of Lee *et al*. (59) in 2004, Khosravi-Zanjani *et al*. (13) in 2014 and the similar study done by Krasaekoopt *et al*. (56) in 2004 who reported that no *Bifidobacterium bifidum *survived in the simulated gastric of pH 1.55 for 15 min. It is worth mentioning that microencapsulation of *B.bifidum* and *B.adolescentis* maintained their viability significantly and increased their survival rate as compared to their free form (*P *< 0.05).

According to Chen *et al. *(49) in 2010 and Borza *et al*. (51) in 2010 disintegration time in gastric juice along with pepsin, was delayed with increasing genipin concentrations. The delayed release of entrapped drugs and proteins within genipin cross-linked hydrogel matrices has been reported by other authors (44-46, 48, 53). Our study represented that genipin cross-linked microcapsules provide the best protection in simulated gastric juice and improve more viability of probiotic baceria in acidic pH of simulated gastric juice. Annan *et al*. (52) in 2008, Borza *et al. *(51) in 2010 and Chen *et al. *(55) in 2007 indicated that the increase in resistance against degradation could be the result of cleavage sites within the microcapsules being changed by the cross-linking action resulting in the reduction of the pore size and limitation of interaction between cells with the gastric juice. Moreover, the authors explained genipin cross-linking action resulting in the inhibition of the enzyme-substrate interaction and this strength structure may reduce the porosity of microcapsules and produce a higher stereohindrance for the penetration of enzymes due to the bulky heterocyclic structure of genipin (48-55). Moreover, it has been reported that presence of different fillers in the structure of calcium alginate capsules increases their resistance under acidic gastric conditions and prevents their rapid disintegration leading to delayed penetration of gastric juice into the microcapsules (5, 10, 13, 20, 23, 25).

The increase in viable counts of bacteria could be attributed to the addition of Hylon, which acts as a filler compound. This may have been due to high levels of amylose in this starch, which led to higher water absorption capacity and consequent enhancement of capsule structure during the gastro intestinal condition. Because starches of high amylose content, such as Hylon, reduce the leakage and water penetration of microcapsules, this structure can demonstrate more strength under adverse conditions, such as acidic pH of simulated gastric juice and hence prolonging time of degradation (25, 27, 30, 31). The low penetration rate of Hylon starch was ex­plained by the presence of endogenous lipids and high-amylose content (30, 31). Crittenden *et al.* (30) in 2001 demonstrated that there is the strong correlation between the binding capacities of the various types of starch and the average surface areas that the different granules, which were different sizes, provided for cell adsorption. They also demonstrated that Hylon maize starch granules have a high ratio of surface area to mass and also a good binding capacity. Therefore, some studies reported the good potential of *bifidobacteria* to adhere to starch for use in microencapsulation technology and for synbiotic applications (25, 27, 30, 31).

**Table 1:** Size and encapsulation yields of different microcapsules

**Probiotic**	**Microcapsule type**	**Mean size of microcapsules (μm)**	**Microencapsulation yield (%)**
*Lactobacillus* strains (*L.casei* and *L. rhamnosus*) *Bifidobacerium* strains (*B.bifidum* and *B.adolescentis*)	Alginate-Hylon- poly-L-lysine	114 ± 2.21	97.24%
	Alginate-Hylon-chitosan	117 ± 3.41	99.31%
	Alginate-Hylon-poly-L-lysine cross-linked with genipin	115 ± 1.81	98.67%
	Alginate-Hylon-chitosan cross-linked with genipin	116 ± 2.24	96.22%
	Alginate-Hylon-poly-L-lysine	113 ± 3.81	99.59%
	Alginate-Hylon-chitosan	118 ± 2.44	96.36%
	Alginate-Hylon-poly-L-lysine cross-linked with genipin	119 ± 1.71	97.29%
Alginate-Hylon-chitosan cross-linked with genipin	114 ± 2.67	96.71%

**Figure 2:**
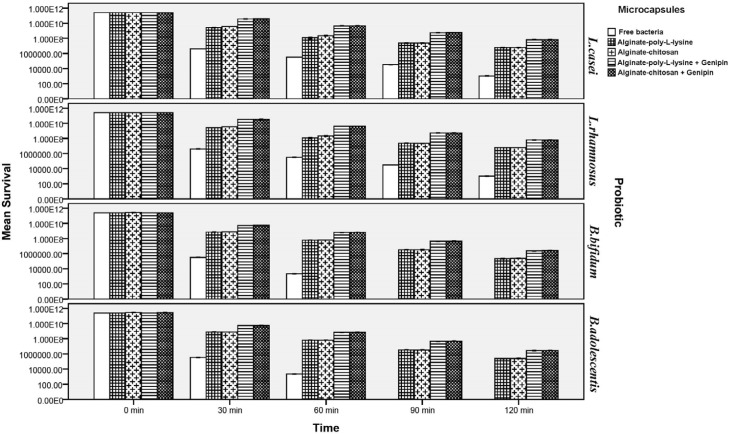
Survival of free and microencapsulated probiotics in simulated gastric juice

**Figure 3:**
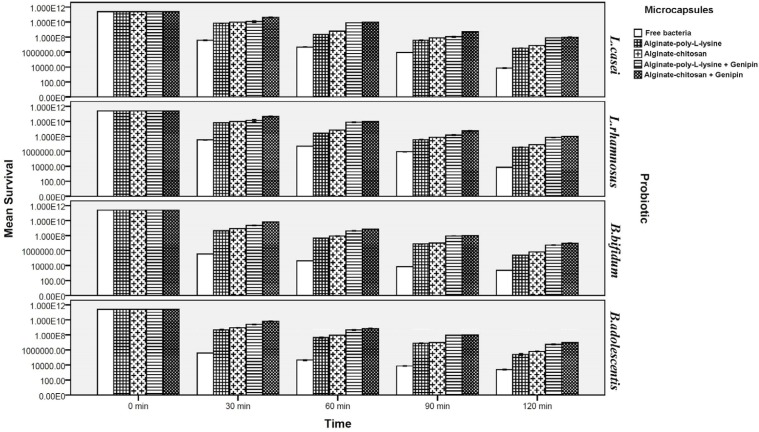
Survival of free and microencapsulated probiotics in simulated intestinal juice

**Figure 4:**
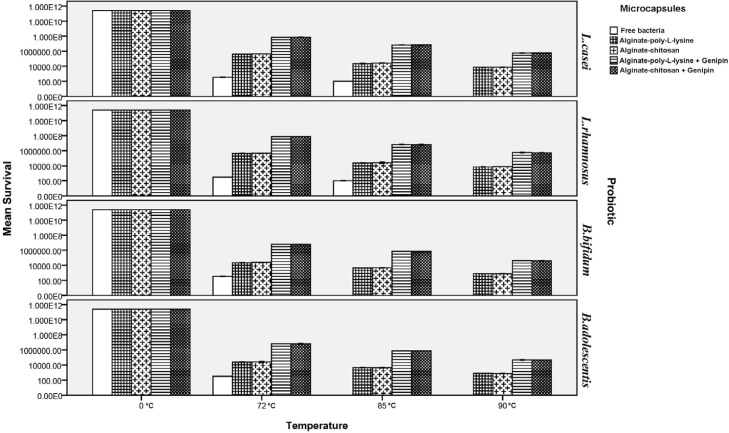
Survival of free and microencapsulated probiotics under heat treatment (72, 85, and 90 °C, 0.5 min).

*Survival of free and microencapsulated bacteria in simulated intestinal juice*

Figure 4 illustrates that the survival of probiotics was lower in intestinal juice and decreased further as the incubation period increased. Viability of microencapsulated probiotics increased significantly as compared to their free form so that viability of cells microencapsulated by chitosan and poly-L-lysine coatings (without a cross-linking agent) increased by about 3.31 and 2.36 log cycles, for *Lactobacillus* strains and approximately 3.45 and 2.27 log cycles for *Bifidobacteria *respectively after 120 min incubation. The loss of viability for cells encapsulated with genipin cross-linked microcapsules was significantly lower in comparison to non-cross-linked microcapsules *(P* < 0.05). It must be mentioned that, as under simulated gastric conditions, there were no significant differences between strains of *L.casei* and *L.rhamnosus*, or between two strains of *B.bifidum* and *B.adolescentis*, with respect to their viability. Cross-linking by genipin in the structure of the capsules significantly improved viability: by about 2.11 and 2.28 log cycle for *Lactobacillus* and *Bifidobacterium* strains respectively after 120 min incubation. However, viability of probiotics with the chitosan and poly-L-lysine coatings differed.

Although both chitosan and poly-L-lysine coatings considerably increased survival rates of the probiotics, survival rates of probiotics microencapsulated with chitosan coating (both cross-linked and not cross-linked by genipin) were significantly higher than those microencapsulated with poly-L-lysine coating (both cross-linked and not cross-linked by genipin). This can be attributed to the greater resistance of chitosan to bile salts and to its resistance to high pH values as compared to poly-L-lysine. This is in good agreement with the results of Krasaekoopt *et al*. (13) in 2004 who indicated that the survival of probiotic bacteria was highly enhanced in gastro-intestinal conditions when encapsulated with alginate-chitosan in comparison with poly-L-lysine coated microcapsules. Ding and shah (39) in 2008 indicated that additional coating of microcapsules with palm oil and poly-L-lysine did little to enhance protection from exposure to bile salt and did not further enhance the protective effects of microcapsules. Many scientists reported that chitosan coating provides the best protection in bile salt solution since an ion-exchange reaction occurred when the microcapsules absorbed the bile salt, therefore the permeability of bile salt into the microcapsules may be restricted (13,14,32-35). Koo *et al*. (60) in 2001 and Chávarri *et al. *(14) in 2010 reported that *Lactobacillus casei *and *Lactobacillus gasseri *microencapsulated in chitosan-coated microcapsules had higher viability than in microcapsules without chitosan coating in bile salt solution.

Our result indicated significantly that genipin cross-linked microcapsules were most effective in protecting probiotic bacteria from simulated intestinal juice (*P* < 0.05). Chen *et al.* (50) in 2009, and Borza *et al. *(51) in 2010 reported that covalent genipin cross-linking is an effective interaction to improving chemical and proteolytic resistance of microcapsules to the intestinal condition. They also indicated that the genipin cross-linked microcapsules remained physically intact after subjected to simulated intestinal condition, and microcapsules with genipin cross-linking appeared robust and largely retained spherical morphology. Furthermore, Khosravi-Zanjani *et al.* (13) in 2014 reported that presence of gelatinized starch together with chitosan increased viability of *L.casei* and *B.bifidum* under simulated intestinal conditions because it delayed penetration of intestinal juice into the microcapsules. Moreover, the improved viability of the probiotics can be attributed to the use of Hylon starch. The protective effect of high amylose maize starch on the bile acid tolerance was measured by Wang *et al*. (25) in 1999. They found that amylomaize promotes the survivability of probiotics by adhesion to starch granules at 0.05% bile acid concentration. Because of the high internally associated structure of Hylon, therefore, it promotes the stability of microcapsules in harsh condition (30, 31). Different studies have shown that microcapsules are better protected in the presence of prebiotics or filler materials and the combination of microcapsules with these compounds not only improve the viability of probiotics but facilitates formation of an integrated structures of capsules (2,11, 13, 14, 17, 19, 24).

*Thermal stability of microcapsules*

Heat treatment of free and microencapsulated probiotics was carried out by exposing them to 72, 85, and 90 °C for 0.5 min to determine their thermal stability. As shown in Figure 5, viability of free probiotics severely declined under heat treatment so that no *Bifidobacteria* that were not microencapsulated remained alive at 85 of 90 °C, and the number of *Lactobacilli* also decreased to zero at 90 °C. However, microencapsulation significantly increased viability of the probiotics so that viability of *Lactobacilli* microencapsulated by chitosan and poly-L-lysine increased by 61.5% on average at 72, 85, and 90 °C, while the corresponding increase for *Bifidobacteria* was about 72.6%. Moreover, thermal resistance of genipin was tested for the first time in this research.

Results indicated that genipin improved viability of the probiotics under the tested heat treatment processes by about 2.01 log cycle for all the tested coatings as compared to capsules without cross-linking. This increase in viability can be attributed to the strengthening of the structures of chitosan and poly-L-lysine by genipin because genipin increases the strength of the walls of the capsules under conditions of high temperature through creating a coherent structure and by forming intermolecular bonds with the amine groups in chitosan and poly-L-lysine (44-49). The current results agree with the findings of Kim *et al.* (61) in 2001 and sabikhi *et al*. (57) in 2010 who reported that *L. acidophilus* bacteria were very sensitive to heat shock and had no growth at 90 °C. Mandal *et al.* (62) in 2006 reported that free cells in distilled water (9.20 log cfu/mL) were drastically reduced to 5.55, 4.93, and 3.98 log cfu/mL on heat treatments at 55, 60, or 65 °C for 20 min, respectively. However, there are no data available at very high temperatures. They also reported that the survival of the encapsulated probiotics might be due to the high additional protection given by starch and high concentration of calcium alginate. Moreover, presence of Hylon starch in the structure of microcapsules can greatly increase their thermal stability. Because of its high amylose content (which results from its high gelatinization temperature), Hylon starch has greater thermal resistance compared to starches with lower amylose content. In addition, the hydrogen bonding between the chains in high amylose structures results in highly internal associations (20, 27, 30, 31).

## Conclusions

The present study has, for the first time, provided information on the survival of four probiotic strains in a novel microencapsulation formulation. Microencapsulation of probiotics in alginate coated by chitosan and poly-L-lysine with the presence of genipin and Hylon as a cross-linker and filler compound, respectively, resulted in better survival of cells after heat treatment and simulated gastrointestinal conditions as compared with free cells. The size of microcapsules was not significantly (*P* > 0.05) influenced in the presence of genipin as a cross-linker. Of the four types of microcapsules in this research, microcapsules cross-linked by genipin provided the best protection for cells and there was no survival of free bifidobacteria in the presence of gastric juice due to its low acid resistance. The presence of genipin as a cross-linker not only increased the viability of probiotics under simulated gastrointestinal conditions but also resulted in higher thermal resistance of the microcapsules produced. Moreover, the combination of chitosan and genipin was the best coating for the viability of probiotics under simulated intestinal conditions. Starch with a high amylose content (with the commercial name of Hylon VII) was used for the first time together with genipin in this research. This combination can improve the structure of the produced microcapsules with regard to resistance to heat or to unfavorable gastrointestinal conditions. Future research must concentrate on techniques and equipment that can be applicable in large-scale commercial operations that are commonly employed in bioactive ingredients, nutraceuticals, food ingredients, and drugs. Moreover, related studies are required to evaluate the development of this novel formula with these probiotics in the gastrointestinal tract using animal models. It is important to note that the positive results of our study may encourage other researchers to investigate other novel coating materials.
